# The long and short of lifespan regulation by Argonautes

**DOI:** 10.1371/journal.pgen.1007415

**Published:** 2018-06-21

**Authors:** Kristen C. Brown, Taiowa A. Montgomery

**Affiliations:** 1 Department of Biology, Colorado State University, Fort Collins, Colorado, United States of America; 2 Cell and Molecular Biology Program, Colorado State University, Fort Collins, Colorado, United States of America; Harvard Medical School, UNITED STATES

MicroRNAs (miRNAs) are short noncoding RNAs that control nearly all aspects of plant and animal development. Each miRNA acts as a sequence-specific guide to direct an Argonaute protein to a target mRNA for silencing [1]. As with most genes, the expression of individual miRNAs fluctuates during development; however, global downregulation of miRNAs often occurs in aged animals [2–6]. Nonetheless, individual miRNAs have important roles in aging and can both shorten and extend lifespan [4, 7]. While studies exploring the roles of miRNAs in longevity have largely focused on individual miRNAs or essential miRNA biogenesis factors, it is possible that different branches of the pathway have distinct roles in regulating core cellular processes associated with aging. In this issue, Aalto and colleagues [8] discover that in *Caenorhabditis elegans* the 2 main arms of the miRNA pathway have opposite roles in regulating lifespan.

Of the approximately 25 Argonautes in C. e*legans*, only 3—*alg-1*, *alg-2*, and *alg-5*—are dedicated exclusively to the miRNA pathway [9–11]. *alg-1* and *alg-2* are largely redundant with one another, and loss of function in either but not both is permissible. *alg-5* appears to define a distinct branch of the miRNA pathway that acts in the germline [11]. Much is known about the roles of the miRNA-associated Argonautes in development, but less is known about their specific functions in adult animals. To better understand the roles of the miRNA-associated Argonautes in postdevelopmental aging, Aalto and colleagues [8] examined the expression of *alg-1* and *alg-2* during the first 5 days of adulthood. ALG-1 levels rapidly declined during aging, while ALG-2 levels remained steady. A recent study by Inukai and colleagues [2] also identified a decline in *alg-1* expression as animals aged and a critical role for *alg-1* in regulating global miRNA levels in aged animals. These results suggest that *alg-1* likely has a central role in aging, while *alg-2* may have a more specialized role.

How does loss of *alg-1* or *alg-2* impact *C*. *elegans*’ longevity? Because of their sequence-relatedness and overlapping roles in many processes, one would predict that ALG-1 and ALG-2 function redundantly in aging as well and thus might act synergistically to control lifespan. Surprisingly, Aalto and colleagues [8] discovered that *alg-1* mutants are short lived, consistent with previous studies [7], whereas *alg-2* mutants are long lived. Aalto and colleagues [8] then asked what effect loss of both *alg-1* and *alg-2* activity has on lifespan. The authors circumvented the lethality of the double mutant by treating *alg-2* mutants with RNAi during a late larval stage just before adulthood. Not surprisingly, *alg-1* RNAi negated the extended lifespan of *alg-2* mutants. Interestingly, *alg-1* depletion led to a greater reduction in lifespan in *alg-2* mutants than in wild-type, suggesting that *alg-1* and *alg-2* have overlapping roles in cellular processes important for healthy aging despite their opposite roles in longevity.

What causes *alg-2* mutants to have extended lifespans while *alg-1* mutants die prematurely? Aalto and colleagues [8] tested whether miRNAs known to extend lifespan are associated with ALG-1 and conversely, if those that shorten lifespan are associated with ALG-2 in aged animals. lin-4 and miR-71, 2 miRNAs whose depletion leads to shortened lifespan [4, 12], were more strongly associated with ALG-1 than with ALG-2 and were depleted in *alg-1* mutants more so than in *alg-2* mutants. While no miRNAs known to affect lifespan associated more strongly with ALG-2 than with ALG-1, miR-239a and miR239b—miRNAs that, when mutated, extend lifespan [4]—were depleted in *alg-2* mutants. Although the results are not black and white, the trend suggests that miRNA specificity plays an important role in the longevity of *alg-1* and *alg-2* mutants. While differences in miRNA binding and temporal or spatial expression of the 2 proteins likely account at least in part for their opposite roles, it is possible that the 2 Argonautes also have distinct biochemical functions from one another. ALG-1 and ALG-2 share approximately 80% amino acid similarity but diverge substantially at their N termini, which could lead to different protein interactions or subcellular localization [13].

**Fig 1:**
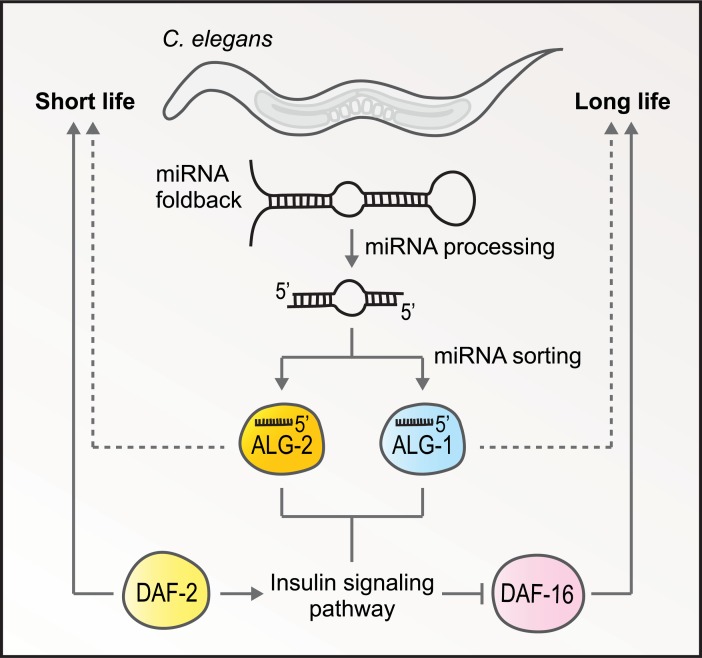
Opposite roles for 2 Argonautes in *C*. *elegans* lifespan regulation. miRNAs are processed from hairpin precursors and sorted between Argonautes, primarily ALG-1 and ALG-2. Loss of *alg-1* activity leads to a shortened lifespan, indicating that ALG-1 normally promotes longevity. Conversely, loss of *alg-2* activity extends lifespan, indicating that ALG-2 acts counter to ALG-1 to limit lifespan. Both Argonautes function at least in part through the insulin signaling pathway involving the insulin/IGF receptor DAF-2, which limits lifespan, and the forkhead transcription factor DAF-16/FOXO, which promotes extended lifespan. It is possible that ALG-1 and ALG-2 also affect longevity via regulation of parallel pathways involved in aging, as indicated by the dashed lines.

Aalto and colleagues [8] next set out to identify the genetic circuits that might underlie the lifespan phenotypes of *alg-1* and *alg-2* mutants. Each of the miRNAs associated with longevity and differentially affected by *alg-1* and *alg-2* exert their function at least in part through the insulin signaling pathway involving the insulin/IGF receptor DAF-2 and the downstream transcription factor DAF-16/FOXO [4, 12, 14]. Many genes involved in insulin signaling, specifically those targeted by DAF-16, were differentially regulated in *alg-1* and *alg-2* mutants. Further linking ALG-1 and ALG-2 to the insulin signaling pathway, the authors discovered that reduced *daf-2* activity extended the lifespans of *alg-1* mutants. In contrast, loss of *daf-16* further reduced the lifespan of *alg-1* mutants, indicating that the role of *alg-1* is not entirely related to DAF-16. This result is not surprising, as the authors identified thousands of genes misregulated in *alg-1* mutants, many of which are not involved in insulin signaling. Loss of both *daf-2* and *alg-2* was additive in lifespan extension, suggesting that *alg-2* may also exert its function in a manner partially independent of insulin signaling (Fig 1). Inactivation of *daf-16*, however, completely suppressed the extended lifespan of *alg-2* mutants, demonstrating a clear link between *alg-2* and *daf-16* in lifespan extension.

Is modulating the miRNA pathway a viable approach for extending lifespan? miRNAs have widespread roles in cellular and organismal aging, although their roles in regulating lifespan are best understood in *C*. *elegans* [15]. It will be important to determine if manipulating the miRNA pathway can extend the lifespans of other species as well. While specific miRNAs could be targeted to extend lifespan in *C*. *elegans*, it is possible that disabling core proteins in the pathway would be more effective than disabling individual miRNAs. Many species contain multiple miRNA-associated Argonautes with overlapping roles. Humans, for instance, contain 4 [1]. Perhaps certain Argonautes could be targeted to extend lifespan in humans and other mammals, although, given their pleiotropic roles in development, it is likely that there would be a cost associated with disrupting any aspect of the miRNA pathway.
